# Bio-waste-derived hydroxyapatite from fish bone, bovine bone, and bovine teeth for enamel remineralization

**DOI:** 10.3389/froh.2026.1894173

**Published:** 2026-09-03

**Authors:** Chawan Karim Jabar, Hadi M. Ismail

**Affiliations:** 1 Department of Pedodontics and Community Oral Health, College of Dentistry, University of Sulaimani, Sulaymaniyah, Iraq; 2 Orthodontics, Department of Orthodontics, College of Dentistry, University of Sulaimani, Sulaymaniyah, Iraq

**Keywords:** bioactivity, bovine bone, bovine teeth, calcium phosphate ratio, fishbone, hydroxyapatite (HAP)

## Abstract

**Objectives:**

To investigate whether calcined fish bone, bovine bone, and bovine teeth can be converted into bioactive hydroxyapatite powders, and whether these materials can function as slurries and promote the remineralization of enamel. The novelty of this study is the production of hydroxyapatite from biowaste-derived bovine bone and bovine teeth powders, and its use as remineralizing agents for demineralized enamel for the first time.

**Methods:**

Eighty-four human premolar enamel specimens were allocated into seven groups (*n* = 12). The physical and chemical properties were done for calcined biowaste materials. Experimental groups were treated with slurries prepared from FiB, BoB, and BoT, while control groups included TM, demineralized enamel, non-protected enamel, and sound enamel. Remineralization was assessed using SEM, EDX, and AFM.

**Results:**

Characterization confirmed the hydroxyapatite-like structures formation with Ca/P ratios varying from 1.55 to 1.64, mesoporous architecture (10–20 nm), and variable crystallinity. The experimental groups had higher calcium content and Ca/P ratio comparing to the negative control group (*p* < 0.05), was very close to that in TM group and sound enamel. The SEM showed in treated enamel surfaces were smoother with clear crystal deposits, especially in the BoT group. Treated enamel surface in the BoT group showed less porosity and a more uniform surface. AFM analysis showed that surface roughness in the BoT and TM groups was significantly reduced (*p* < 0.05), such as Sq value in TM group decreased from 33.6 nm to 10.2 nm.

**Conclusion:**

The calcined biowaste-derived HAp materials showed an improvement in the remineralization of enamel, and the bovine tooth-derived HAp had the best overall performance.

## Introduction

Enamel demineralization is a common dental condition that first appears as white spot lesions (WSLs) and, if not treated promptly and properly, can develop into cavities (tooth decay). Tooth decay is the most widespread disease globally. The estimated global prevalence rate for children is 30%–50%, whereas for adults it is 20%–45% (1). Early-stage intervention through remineralization is essential to restore mineral loss and prevent lesion progression.

Enamel remineralization is a process where calcium and phosphate ions from an external source are provided to the tooth surface, and ions diffuse into voids in demineralized enamel and are gradually filled, resulting in a net gain in mineral content. Mineral deposition refers to the formation of new mineral within the lesion, which increases the density and the structural integrity of the lesion. For this to occur, calcium and phosphate ions need to be supplied to the lesion in amounts that allow their precipitation as solid mineral phases (2). In the past 20 years, a number of novel synthetic calcium phosphate (CaP) systems have been developed to support this process, including tricalcium phosphate (TCP), amorphous calcium phosphate (ACP), casein phosphopeptide-amorphous calcium phosphate (CPP-ACP), functionalized *β*-tricalcium phosphate (fTCP), calcium sodium phosphosilicate, and casein phosphopeptide-amorphous calcium fluoride phosphate (CPP-ACFP), and each material serves as a reservoir of bioavailable calcium and phosphate ions. These materials are designed to enhance enamel remineralization and inhibit the progression of early carious lesions (3, 4).

However, they are time-consuming, costly, and ecologically taxing. In contrast, in recent years, CaPs from natural sources have been increasingly used as replacement materials for synthetic CaPs, providing eco-friendly, low-cost alternatives to hydroxyapatite production via calcination (5–7).

The biowaste materials such as fishbone, bovine bone, and teeth materials are strongly influenced by their chemical composition, especially the calcium-to-phosphate (Ca/P) ratio, crystallinity, and structural phase (5–10). Over 190,000 tons of bovine products and 91 million tons of fish are consumed annually, generating substantial amounts of biological waste, including bones and teeth. The by-products are suitable raw materials for the manufacture of calcium phosphate through calcination (5, 9).

While significant research has been conducted on the use of fishbone extracts to enhance enamel remineralization, these studies confirmed that the calcium to phosphate ratio (Ca/P ratio) derived from bones of different types of fish was suitable for remineralization (approximately 1.63, 1.6, and 1.71 molar for carp, sardine, and tuna fishbone, respectively), that contain hydroxyapatite (HA), and benefecial trace elements that support remineralzation (11). Several studies have isvestigated different methods for the extraction of hydroxyapatite HA from different animal bone species as a natural resource. However, no study applied it as a remineralization agent (4).

The present study was undertaken with two primary objectives: (1) to synthesize hydroxyapatite-like calcium phosphate powders from calcined biowaste sources, such as fish bone, bovine bone, and bovine teeth, and verify their successful synthesis through several physicochemical characterisation methods, including FTIR, XRD, EDX, SEM, BET/BJH, and DSC, and (2) to assess the enamel remineralisation potential of these powders, when applied as slurries to artificially demineralised enamel, by employing both surface-based and compositional analyses. We hypothesised that all three biowaste-derived powders would possess apatite-like properties, and would bring about measurable improvements in enamel remineralisation compared to negative controls, with effects approaching those of Tooth Mousse, while the null hypothesis was that there would be no statistically significant differences in remineralisation outcomes between enamel treated with the experimental powders and the control groups.

## Materials and methods

### Study design and ethical approval

This experimental *in vitro* study evaluated the remineralization potential of bioactive materials derived from carb fish bone (FiB), bovine bone (BoB), and bovine teeth (BoT), compared with a commercially available agent (Tooth Mousse). Enamel remineralization was assessed by analyzing surface morphology, elemental composition, and surface roughness using scanning electron microscopy (SEM), energy-dispersive x-ray spectroscopy (EDX), and atomic force microscopy (AFM).

This study was approved by the ethical committee of the College of Dentistry, University of Sulaimani (COD-EC-24-0030) and the extracted premolar teeth were collected from various dental clinics. The teeth were de-identified because the college biosafety and ethics guidelines were observed during handling of the materials. The fish bone, cow bone, and cow teeth were wastes from the local seafood stores and butchers in Sulaymaniyah, Iraq, thus no animal was sacrificed for this study.

### Sample collection

A total of eighty-four human premolar teeth extracted for orthodontic purposes were collected. Teeth with cracks, caries, restorations, fluorosis, or structural defects were excluded. All the teeth were cleaned after extraction to remove any debris and remaining soft tissue, and they were stored in 0.1% thymol solution at 4 °C to prevent microbial growth till used.

### Specimen preparation

Each tooth was sectioned at 2 mm from cementoenamel junction to obtain standardized enamel specimens using a low-speed diamond saw under water cooling. The enamel surfaces were flattened and polished using silicon carbide abrasive papers of increasing grit sizes (e.g., 600–1,200 grit) to obtain a uniform and smooth surface. The cut surfaces were embedded in the mold, then stored in a moist environment at 4 °C until analysis (12, 13). Specimens were then rinsed with distilled water and air-dried. The experimental area for EDX, SEM, and AFM analysis, the specimens were embedded in acrylic resin, then the surrounding enamel surface were coated with nail varnish (acid resistant), leaving 5*5 mm enamel window on the buccal surface.

### Group allocation

The enamel samples were randomly allocated into seven groups (*n* = 12/group**)** as follows:

1. FiB group – treated with fish bone-derived material.
2. BoB group – treated with bovine bone-derived material.
3. BoT group – treated with bovine teeth-derived material.
4. TM group Positive control – treated with Tooth Mousse.
5. Negative control (non-protected) – untreated enamel exposed to the oral simulation environment.
6. Negative control (demineralized) – demineralized enamel with no treatment.
7. Sound enamel group – intact enamel with no demineralization or treatment.
8. Each group was further subdivided into two equal subgroups (*n* = 6) for analysis by SEM/EDX and AFM, respectively.

### Demineralization protocol

Except for the sound enamel group, specimens were exposed to a demineralization process using an acidic solution (0.05 M lactic acid, pH 4.5) for 96 h at 37 °C to simulate early enamel lesions. After demineralization, specimens were rinsed with distilled water and stored in artificial saliva until further treatment**.**

### Preparation and characterization of bioactive materials

Carp fish bones, bovine bone, and bovine teeth were cleaned and prepared prior to calcination. Fish and bovine bones were boiled at 100  °C for 1.5 h, dried overnight, then treated with acetone (1:50 ratio) for 2 h, rinsed in distilled water, and air-dried (4, 14, 15). Bovine teeth were cleaned through repeated water washing, steam cycles, and sunlight exposure for 2 days, followed by removal of roots at the cementoenamel junction (4). All samples were then crushed into fine powder. All samples were then ground with the use of laboratory grinder until the visually obtained homogeneous powder.

Thermal calcination was performed using a muffle furnace. Fish bone was calcined at 950  °C for 2 h, bovine bone at 750  °C for 6 h, and bovine teeth using a two-stage process: 735  °C for 1 h followed by 1,000  °C for 1 h (the heating rate of 10  °C/min). All samples were cooled gradually in the furnace to room temperature to prevent structural damage (14, 15).

The calcined materials were characterized to confirm phase composition and physicochemical properties.

### Characterization of the calcined powder

1. Fourier Transform Infrared Spectroscopy (FTIR) (Thermo Science, Nicolet iS10 spectrometer, UK), The spectra were collected at 2 cm^−1^ resolution from 400 to 4,000 cm⁻¹ was used to recognize the functional groups. The phosphate (PO₄³⁻), hydroxyl (OH⁻), and carbonate (CO₃²⁻) groups absorption bands were estimated to confirm hydroxyapatite formation and evaluation of sample purity was based on the distinctive vibrational modes of calcium phosphate.
2. Energy-dispersive x-ray spectroscopy (EDX), the S2 PUMA system (Bruker, Germany) was used to determine elemental composition and Ca/P ratio.
3. x-ray diffraction (XRD) analysis (Cu-K*α*, 2*θ* = 5°–69°) was performed to assess crystalline structure (PANalytical X'Pert PRO diffractometer, Almelo, Netherlands), and crystallite size was determined using the Scherrer equation (T=Kλ/βcosθ)
  Where:
  3.1. **T** is the average crystallite size,
  3.2. **K** is the shape factor (usually 0.9),
  3.3. ***λ*** is the x-ray wavelength,
  3.4. **β** is the full width half maximum (FWHM) of the selected diffraction peak in radians,
  3.5. **θ** is the Bragg angle.

The FWHM values were evaluated using the (005) reflection peak (16).

4. Differential scanning calorimetry (DSC 131evo, Setaram, France) was conducted to evaluate thermal behavior. Representative samples of accurately weighed masses to within 15–20 mg were encapsulated with care in hermetic aluminum crucibles to preserve integrity throughout the high-temperature scan. An inert reference was established using an identical, empty aluminum crucible in each experiment. It was a single dynamic heating ramp increasing the temperature from 40  °C to an end temperature of 600  °C with a constant rate of 25  °C/min. The program was developed to identify the characteristic temperature regimes of endothermic and exothermic processes, including glass transitions, melting, crystallization, and thermal decomposition. The equipment's built-in heat flow signal was measured continuously and subsequently normalized with respect to respective sample mass, with results given in units of microvolts (μV) to allow direct comparison.
  Later data acquisition and processing, plotting and analysis of resulting thermograms to identify onset, peak, and end temperatures for transitions detected, were done using the TA Universal Analysis 2,000 dedicated software package.
5. Surface area, porosity, and pore size distributions (N2 physisorption at 77 K) were determined using Brunauer–Emmett–Teller (BET) analysis (automatic Quantachrome gas sorption analyser, USA), with specific surface area calculated by the Brunauer–Emmett–Teller (BET) method.
6. Scanning electron microscopy (SEM) was used to evaluate particle morphology, size, and distribution, using FEI Quanta 450 Scanning Electron Microscope (FEI, USA). Aluminum stub was used to support the sample and sputter-coated with a silver film of approximately 200 Å thickness for enhancing the resolution. Image analysis was performed using the ImageJ software package, which was provided by the National Institutes of Health, USA (17–19).

### Preparation of demineralization and remineralization solution

The demineralization solution was prepared using CaCl₂ (2.2 mM), NaH₂PO₄ (2.2 mM), and acetic acid (0.05 M), with the pH adjusted to 4.4 using 1 M KOH (20).

The remineralization solution was prepared using CaCl₂.2H₂O (1.5 mM), KH₂PO₄ (0.9 mM), KCl (150 mM), and 4-(2-hydroxyethyl)-1-piperazineethanesulfonic acid (HEPES) (20 mM), with the pH adjusted to 7.0 with the use of 1 M KOH. This solution was formulated to simulate the ionic composition of natural saliva and was fluoride-free (21).

### Preparation of slurry

The calcined powders of FiB, BoB, and BoT were mixed with a remineralization solution for 5 min to obtain a homogeneous slurry at a ratio of 1:3 (1 g powder: 3 mL liquid), achieving a clinically applicable consistency. Similarly, the Tooth Mousse was obtained by mixing 1 g of paste with 3 mL of remineralization solution, following the same proportion (22).

### Treatment protocol

Specimens allocated for demineralization were immersed in 15 mL of freshly prepared demineralizing solution for 96 h, and afterwards, specimens were rinsed in deionized water for 10 s before treatment. The experimental materials were applied to the enamel surfaces with a microbrush for 5 min, and then specimens were briefly immersed in deionized water to increase ionic exchange (10–12 s).

Following application, the specimens were rinsed and immersed in 20 mL of remineralization solution per specimen for 21 h at 37  °C in an incubator. Subsequently, they were rinsed again and immersed in 40 mL of demineralization solution for 3 h at 37  °C. This cycle was followed by the second application of experimental remineralization agents (13, 23).

The pH of the solution was measured daily using a pH meter before treatment. The demineralization solution was replaced every 3 days, and the remineralization solution every 2 days. This continued for 10 days.

Forty-two specimens (*n* = 6 per group) were prepared for SEM and EDX analysis. Specimens were dehydrated, mounted on aluminum stubs, and sputter-coated with gold. SEM was used to evaluate surface morphology at appropriate magnifications, while EDX analysis was performed to quantify elemental composition, particularly calcium (Ca), phosphorus (P), and their ratio.

### Surface morophology and elemental analysis (SEM/EDX)

Enamel specimens were evaluated before and after treatment using scanning electron microscopy (SEM) with energy-dispersive x-ray spectroscopy (EDX), and with the SEM-EDX system, the elemental composition of the enamel surface, including calcium (Ca), phosphorus (P), oxygen (O), and chloride (Cl), was measured (Bruker Nano GmbH, Berlin, Germany).

For morphological evaluation, specimens were rinsed with deionized water, air-dried, mounted on aluminum stubs, and coated with a conductive layer prior to imaging. Surface topography was qualitatively assessed using SEM (Bruker Nano GmbH, Berlin, Germany).

### Surface rughness analysis (AFM)

The enamel specimens surface roughness was evaluated prior and after treatment using atomic force microscopy (AFM) (TT-2 AFM Workshop, USA). Specimens were mounted on the AFM stage, and five different regions per specimen were scanned over an area of 2 × 2 µm. The root mean square roughness (Sq) and arithmetic mean height (Sa), were recorded as surface roughness parameters. Image acquisition and analysis were performed using the AFM control software.

### Statistical analysis

Statistical analysis was performed using GraphPad Prism (version 10.3.1, GraphPad Software, USA). The composition of the enamel and surface roughness parameters were compared among the experimental groups (FiB, BoB, BoT), the positive control (Tooth Mousse) and negative controls (non-protected demineralized enamel and sound enamel) because the distribution of the data was tested by the Shapiro–Wilk test. Data that followed the assumptions of normality were subjected to one-way ANOVA and then to Tukey's *post hoc* test for multiple comparisons, while data that did not follow the assumptions of normality were subjected to the Kruskal–Wallis test. The changes in the AFM measurements before and after treatment within each group were evaluated using paired t-tests, and a *p*-value < 0.05 was considered statistically significant.

## Results and discussion

The current study examined the potential of FiB, BoB, and BoT as green sources of calcium phosphate for use as an enamel remineralization agent.

The differences in the particles' morphology, structure, elemental composition, crystallinity, and size, as well as their physicochemical properties, were assessed using a combination of FTIR, EDX, XRD, DSC, BET, and SEM. After applying the materials as experimental remineralization agents to the enamel surface, the surface was analyzed.

### Results of characterization of bioactive materials

#### Functional groups: FTIR analysis

**Figure 1:**
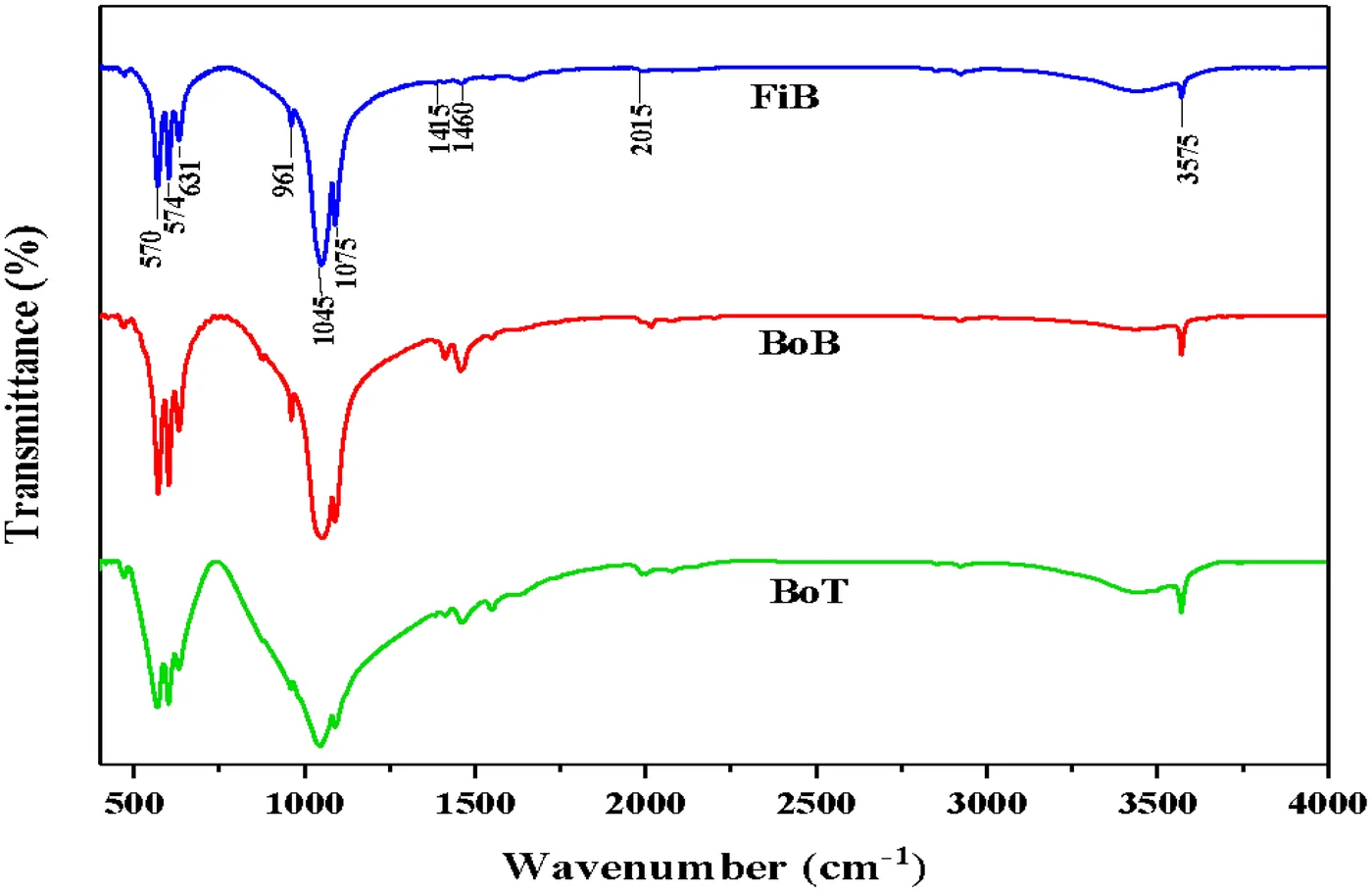
Functional groups in the biowaste materials, fish bone (FiB), bovine bone (BoB), and bovine teeth (BoT).

FTIR analysis confirmed the appearance of characteristic functional groups associated with hydroxyapatite in the experimental materials (Figure 1). The phosphate (PO₄³⁻) groups were identified within the range of 1,090–960 cm⁻¹, with additional bands at 569 and 601 cm⁻¹ corresponding to non-apatitic and acid phosphate components.

Carbonate (CO₃²⁻) substitution within the apatite structure was indicated by bands observed around 580 and 631 cm⁻¹. Additionally, absorption bands corresponding to amine (N–H) and amide (C = O) groups were detected at 3,445–3,570, 1,460, and 1,550 cm⁻¹, suggesting the presence of residual organic components.

Overall, the FTIR spectra confirmed the configuration of a hydroxyapatite-like structure in all experimental materials.

#### Elemental composition: energy-dispersive x-ray spectroscopy (EDX)

Energy-dispersive x-ray spectroscopy (EDX) analysis confirmed that the elemental composition of the calcined materials was consistent with hydroxyapatite (Table 1). The presence of different elements, including calcium, phosphorus, and other trace elements, enhances the bioactivity of the materials. Among fishbone, bovine bone, and bovine teeth, the amounts of calcium (Ca) were 57.76, 58.19, and 58.63, and the amounts of phosphorus (P) were 36.93, 37.35, and 35.75, respectively, whereas the Ca/P ratios were 1.56 and 1.55, and 1.64 moles, respectively.

**Table 1:** The percentage composition of elements of the fishbone (FiB), bovine bone (BoB), and bovine teeth (BoT) using EDX. .

No.	Elements	FiB	BoB	BoT
1.	Ca (%)	57.76	58.19	57.28
2.	*P* (%)	36.93	37.35	37.57
3.	Ca/p ratio (molar)	1.56	1.55	1.53
4.	Si (%)	2.35	2.07	2.2
5.	Mg (%)	1.22	0.93	1.17
6.	K (%)	0.16	0.06	0.05
7.	Al2 (%)	0.75	0.62	0.79
8.	Cl (%)	0.03	0.01	0.15
9.	Fe2 (%)	0.05	0.04	0.03
10.	Sc2 (%)	0.57	0.61	0.55

Trace elements, including silicon (Si), magnesium (Mg), aluminium (Al), potassium (K), chloride (Cl), iron (Fe), and scandium (Sc), were evaluated and are known to impact the bioactivity of the materials.

#### Crystalline phases: XRD

X-ray diffraction (XRD) analysis was performed to estimate the crystalline texture of the calcined materials (Table 2). Characteristic diffraction peaks were observed at 2*θ* values of 25.86° for fish bone (FiB), 31.81° for bovine bone (BoB), and 31.88° for bovine teeth (BoT), confirming the presence of a hydroxyapatite phase.

**Table 2:** Crystal size, 2theta values, and FWHM for hydroxyapatite obtained from fish bone, bovine bone, and bovine tooth.

Sample Name	2*θ* (Degree)	FWHM	Crystallite Size (nm)
Fishbone	25.86	0.0984	83.2
Bovine bone	31.81	0.1378	59.9
Bovine teeth	31.88	0.1574	52.5

The full width at half maximum (FWHM) values were 0.0984, 0.1378, and 0.1574 for FiB, BoB, and BoT, respectively. Crystallite size was different between the materials, and the largest average crystallite size was obtained for FiB (83.2 nm) followed by BoB (59.9 nm) and BoT (52.5 nm), which is in accordance with the degree of crystallinity.

**Figure 2:**
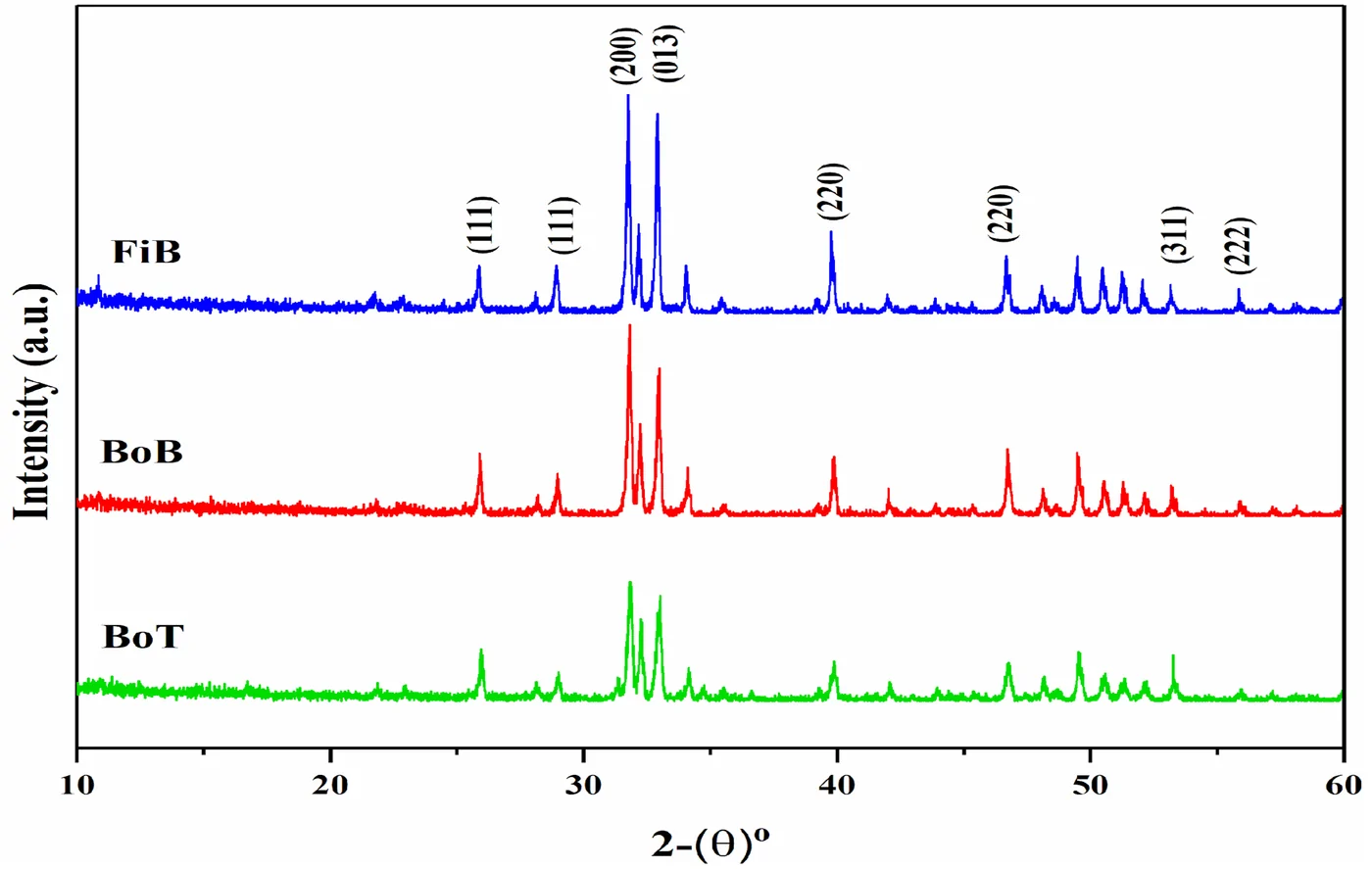
XRD patterns for the calcined bio-waste samples: FiB, BoB, and BoT, and were analyzed to determine their composition.

The x-ray diffraction of calcined raw experimental materials at different temperatures, as shown in Figure 2, showed that the highest FWHM value of 0.1574 for BoT indicates that it has the broadest diffraction peaks, while FiB has the narrowest diffraction peaks with an FWHM value of 0.0984. The broader peaks observed for BoT indicate smaller crystallite size, while the narrower peaks observed for FiB indicate larger crystallites, which is in agreement with the crystallite size calculated using the Scherrer equation. The major diffraction planes are (300), (211), and (112) with the diffraction angles (2θ) of approximately 32.3°, 31.8°, and 32.9°, respectively.

On comparing the experimental XRD patterns with the standard reference patterns of hydroxyapatite (JCPDS/PDF No. 09-0432) and beta-tricalcium phosphate (beta-TCP; JCPDS/PDF No. 09-0169), the main diffraction peaks of all calcined bio-waste samples were found to be in good agreement with the peaks of hydroxyapatite, and no peaks corresponding to beta-TCP or other calcium phosphate secondary phases were detected separately or distinctly. This may indicated that hydroxyapatite was the major crystalline phase in calcined FiB, BoB and BoT samples; however, it was not possible to rule out that very small amounts of secondary phases could be present below the detection limit of XRD or that the peaks could be overlapping with the hydroxyapatite reflections.

### Thermal stability using differential scanning calorimetry (DSC)

**Figure 3:**
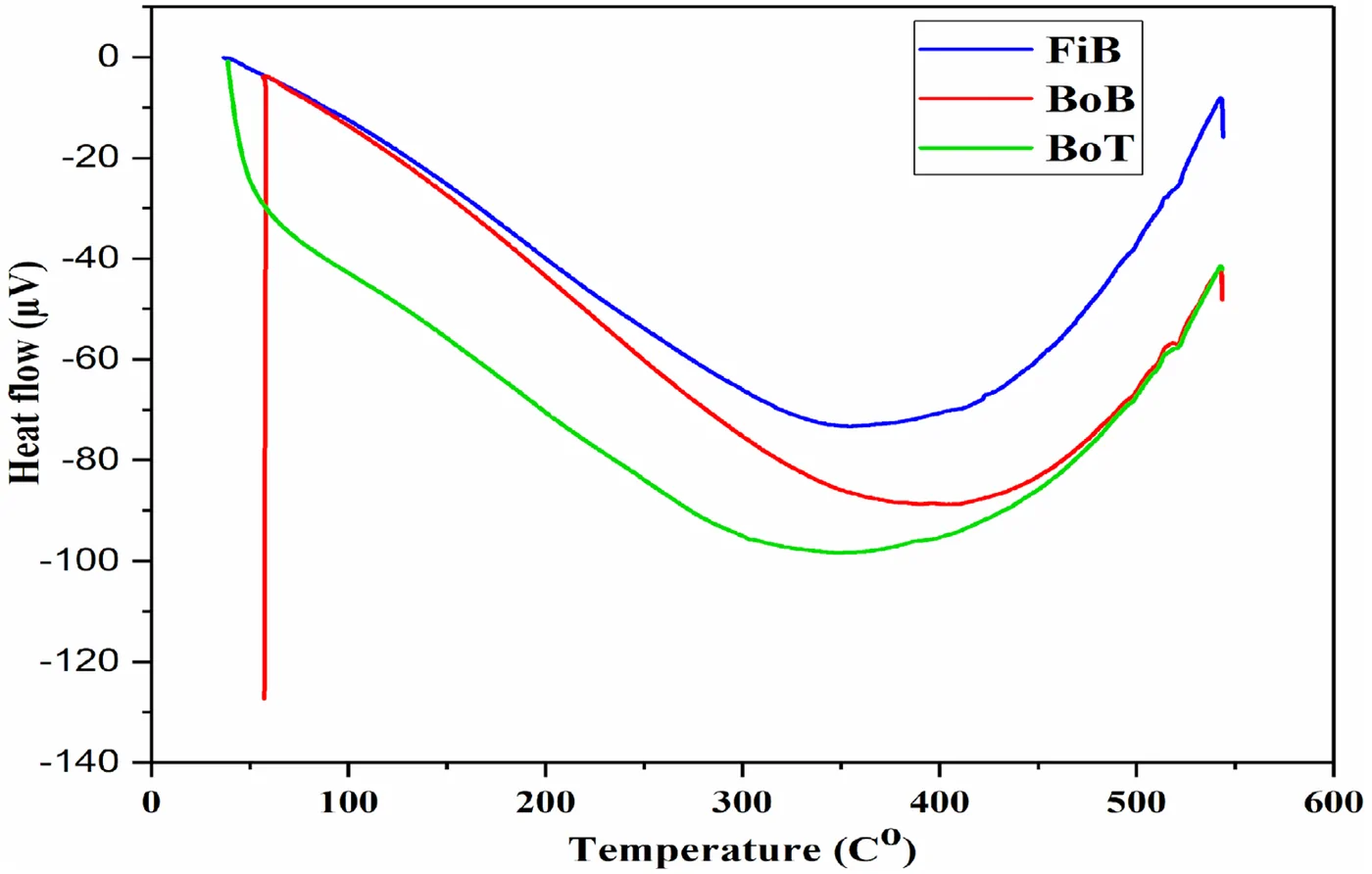
Thermal behavior of FiB, BoB, and BoT was evaluated in terms of heat capacity as a function of temperature using DSC.

The differential scanning calorimetry (DSC) showed different thermal behaviors of the materials (Figure 3). The phase transition temperature was observed at approximately 348  °C for fish bone (FiB), 400  °C for bovine bone (BoB), and 340  °C for bovine teeth (BoT), and the initial transition temperature was between 38.86  °C and 57.38  °C, while the end temperature was about 543  °C for all samples. The phase transition occurred first in BoT, which means that BoT had the lowest thermal stability compared with FiB and BoB.

### Surface area analysis and pore size distribution using Brunauer–Emmett–Teller (BET)

**Figure 4:**
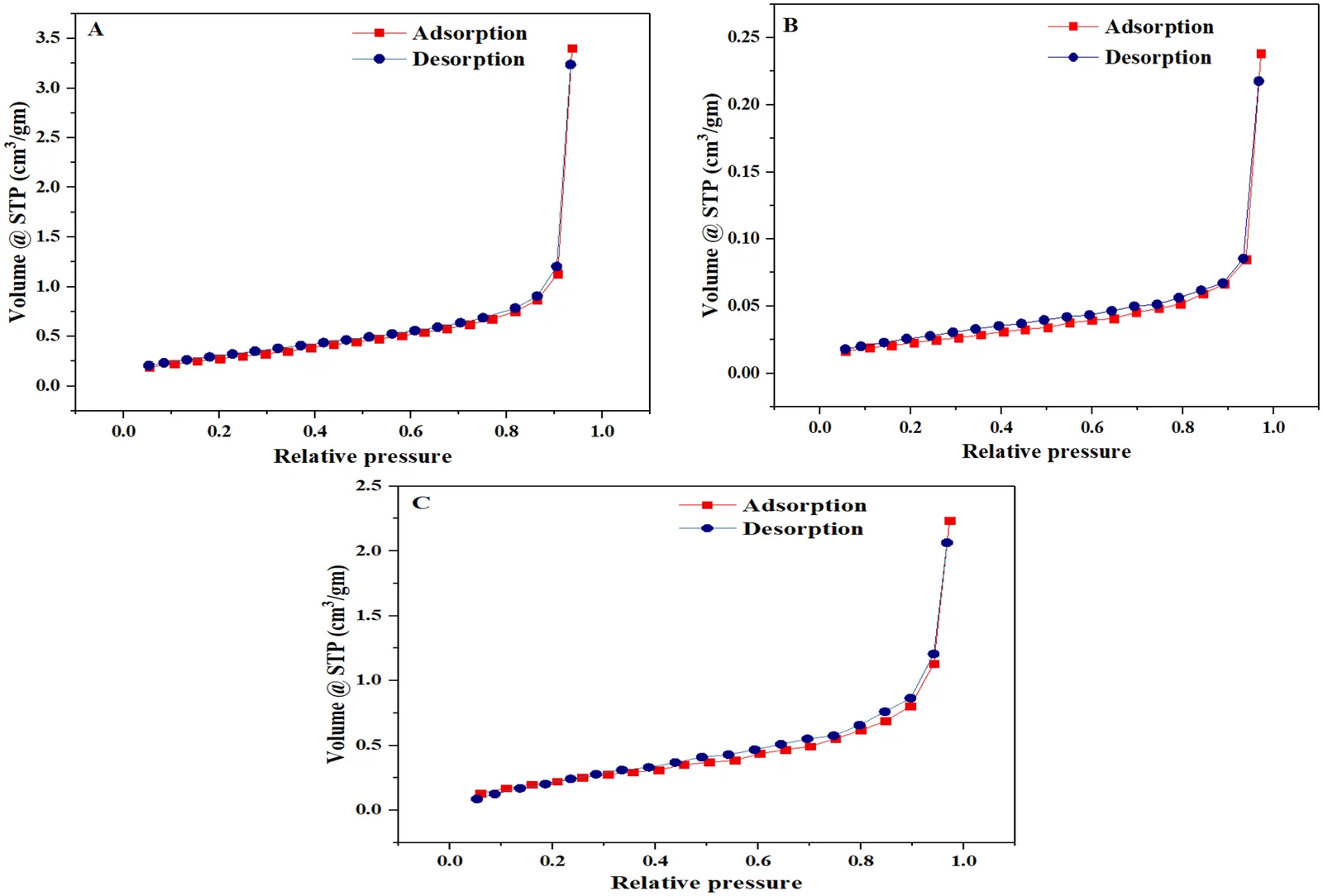
Nitrogen adsorption–desorption isotherms of calcined bio-waste-derived materials, including **(A)** fishbone (FiB), **(B)** bovine bone (BoB), and **(C)** bovine teeth (BoT), show type IV isotherms with a clear hysteresis loop, indicating the mesoporous nature of the materials.

BET analysis was conducted to confirm that the calcium phosphate materials extracted from the sources studied here are mesoporous (Table 3). The identical surface area of the materials was between 0.3 and 2 m²/g, and the highest specific surface area was observed for the extracted materials from FiB and BoT, while the lowest was observed for the extracted material from BoB. The total pore volume was higher for the materials extracted from FiB and BoT (0.01 cm³/g) than that extracted from BoB (7.5 × 10⁻⁴ cm³/g), and the average pore diameter of extracted materials, calculated from Barrett-Joyner-Halenda (BJH) adsorption–desorption data, showed that the materials have pores ranging from 10 to 20 nm, thus confirming their mesoporous nature. All calcium phosphate samples showed type IV adsorption–desorption isotherms with a clear hysteresis loop, which is the typical characteristic of mesoporous materials and evidence of capillary condensation occurring in the pores (Figure 4).

**Table 3:** BET and BJH textural parameters of FiB, BoB, and BoT.

Sample	BET surface Area m^2^/g	BJH Avg. Pore diameter (nm)	Total Pore Volume (cm^3^/g)
Fishbone	1–2	12–15	0.007–0.01
Bovine bone	0.3	10–15	7.5 × 10^–4^
Bovine teeth	1–2	10–20	0.01

### Morphology and microstructure: scanning electron microscopy SEM

**Figure 5:**
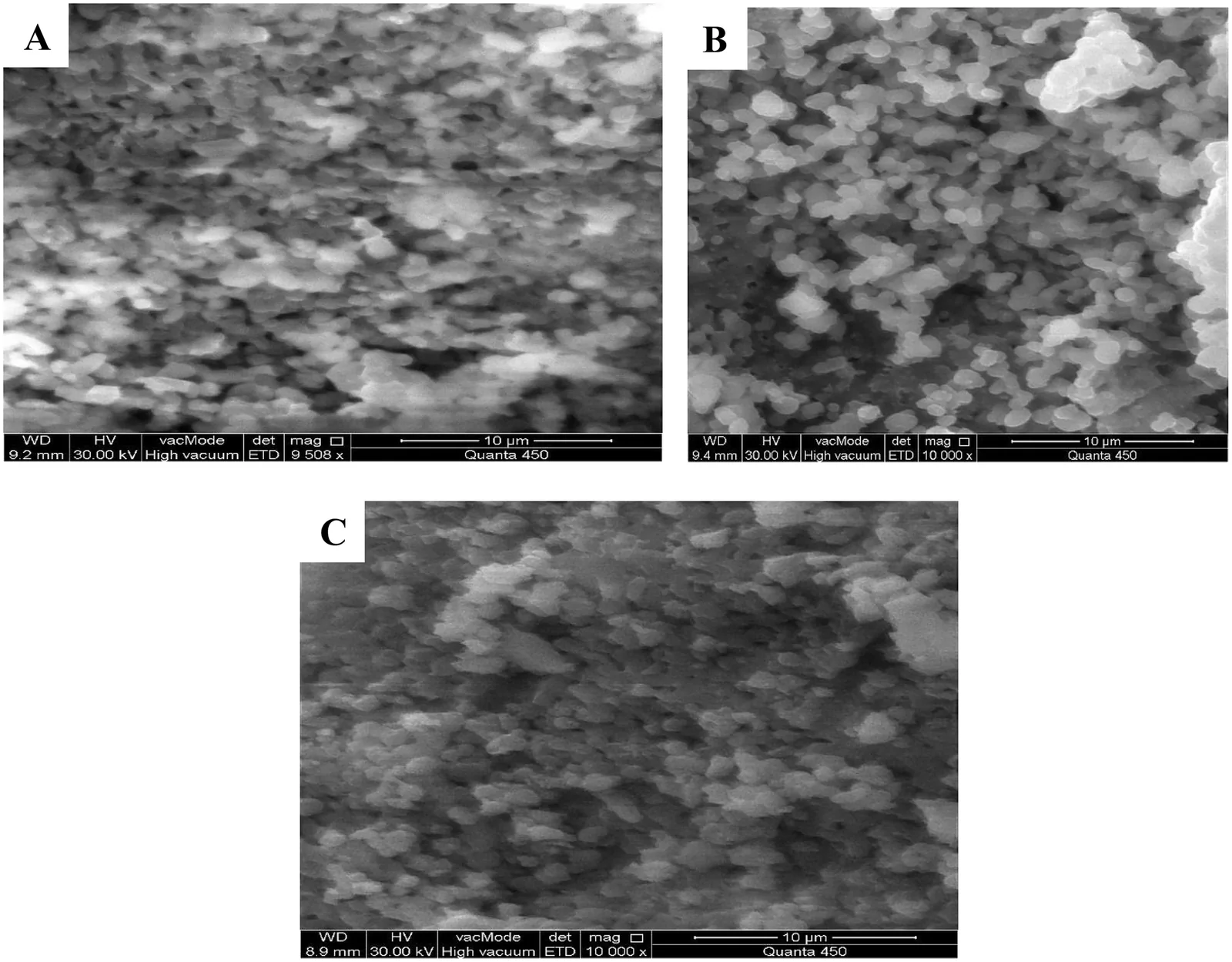
Particle morphology of the calcined bio-waste materials, including **(A)** FiB calcined at 980  °C, **(B)** BoB calcined at 750  °C, and **(C)** BoT calcined at 735  °C for 1 h and then 1,000  °C for 1 h and 10 min.

**Figure 6:**
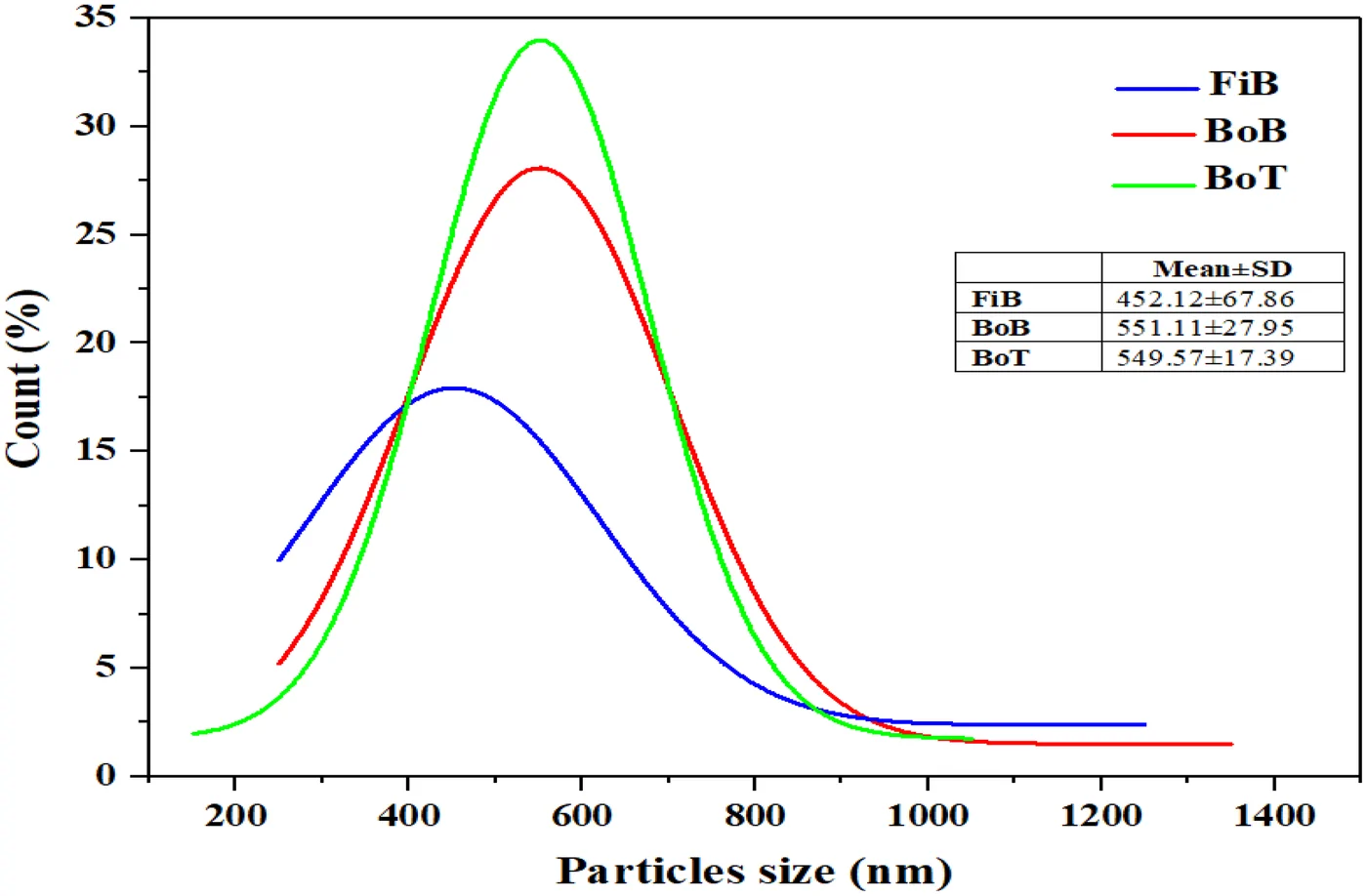
Particle size distribution of calcined bio-waste-derived materials, including fish bone (FiB), bovine bone (BoB), and bovine teeth (BoT), with particle sizes in nm.

Morphological analysis and microstructure of the calcined components by SEM (Figures 5, 6) show great differences in the particle shape, size, and porosity between the groups. The morphology of the calcined FiB was exhibited an irregular, rough surface with interconnected surface voids, with grains sintered and surfaces rough and amorphous, and the pore diameters of FiB ranged between 416.8 and 527.3 nm. The average particle size of FiB was 450.34 ± 68.40 nm, and the porosity was 2.43%. The morphology of the calcined BoB was characterized by a denser morphology and a more granular and porous structure with fewer intergranular voids than the other groups, and the particle sizes of BoB varied between 247.3 and 845.1 nm. The average particle size of BoB was 551.03 ± 28.17 nm, and the porosity was 3.48%. The morphology of the calcined BoT was characterized by a partially sintered and loosely aggregated crystalline structure, and the particle sizes of BoT were relatively uniform, ranging between 339.8 and 373.2 nm. The average particle size of BoT was 549.93 ± 17.08 nm, with the porosity of 2.33%. In summary, the FiB calcined powder had the most porous and irregular architecture, the BoB calcined powder had a more compact and dense architecture, and the BoT teeth calcined powder had a more uniform and homogeneous crystalline arrangement.

### Effect of bioactive materials on enamel composition and surface characteristics

#### Energy-dispersive x-ray spectroscopy (EDX)

As shown in Table 4, there were statistically significant differences in the elemental composition of enamel (mass %) between the study groups. The Ca content in the FiB, BoB, and BoT groups was significantly higher than that of the non-protected demineralized enamel (negative controls; *p* < 0.05), and the P content in the experimental groups was significantly lower than in the negative controls (*p* < 0.05). The Ca/P ratio was significantly increased in all experimental groups when compared to the negative controls (*p* < 0.05), indicating increased mineral deposition; however, there were no statistically significant differences in the Ca, P, and Ca/P ratio between the experimental groups, positive control (Tooth Mousse), and sound enamel (*p* > 0.05). Moreover, there were no statistically significant differences in the content of Cl and O between the experimental and control groups.

**Table 4:** Elemental composition (percentage by mass) of enamel in the experimental and control groups.

Groups	% Ca	% P	Ca/P ratio	% Chloride	% Oxygen
FiB	28.77 ± 0.73	0.51 ± 0.21	2.42 ± 0.15	0.71 ± 0.57	58.6 ± 0.61
BoB	27.89 ± 1.86	1.06 ± 0.43	2.25 ± 0.32	0.48 ± 0.14	59.13 ± 0.79
BoT	27.75 ± 2.14	1.14 ± 0.46	2.24 ± 0.38	0.45 ± 0.11	59.28 ± 1.01
TM	28.79 ± 1.23	0.69 ± 0.28	2.41 ± 0.23	0.36 ± 0.05	58.83 ± 0.49
Non-Protected	25.1 ± 1.53	0.66 ± 0.27	1.76 ± 0.18	0.47 ± 0.12	60.12 ± 1
Demineralized	25 ± 0.56	0.36 ± 0.14	1.74 ± 0.06	1.18 ± 1.56	59.51 ± 1.43
Sound	26.74 ± 1.29	0.69 ± 0.28	2.07 ± 0.21	0.44 ± 0.1	59.86 ± 0.76

#### Scanning electron microscope (SEM)

**Figure 7:**
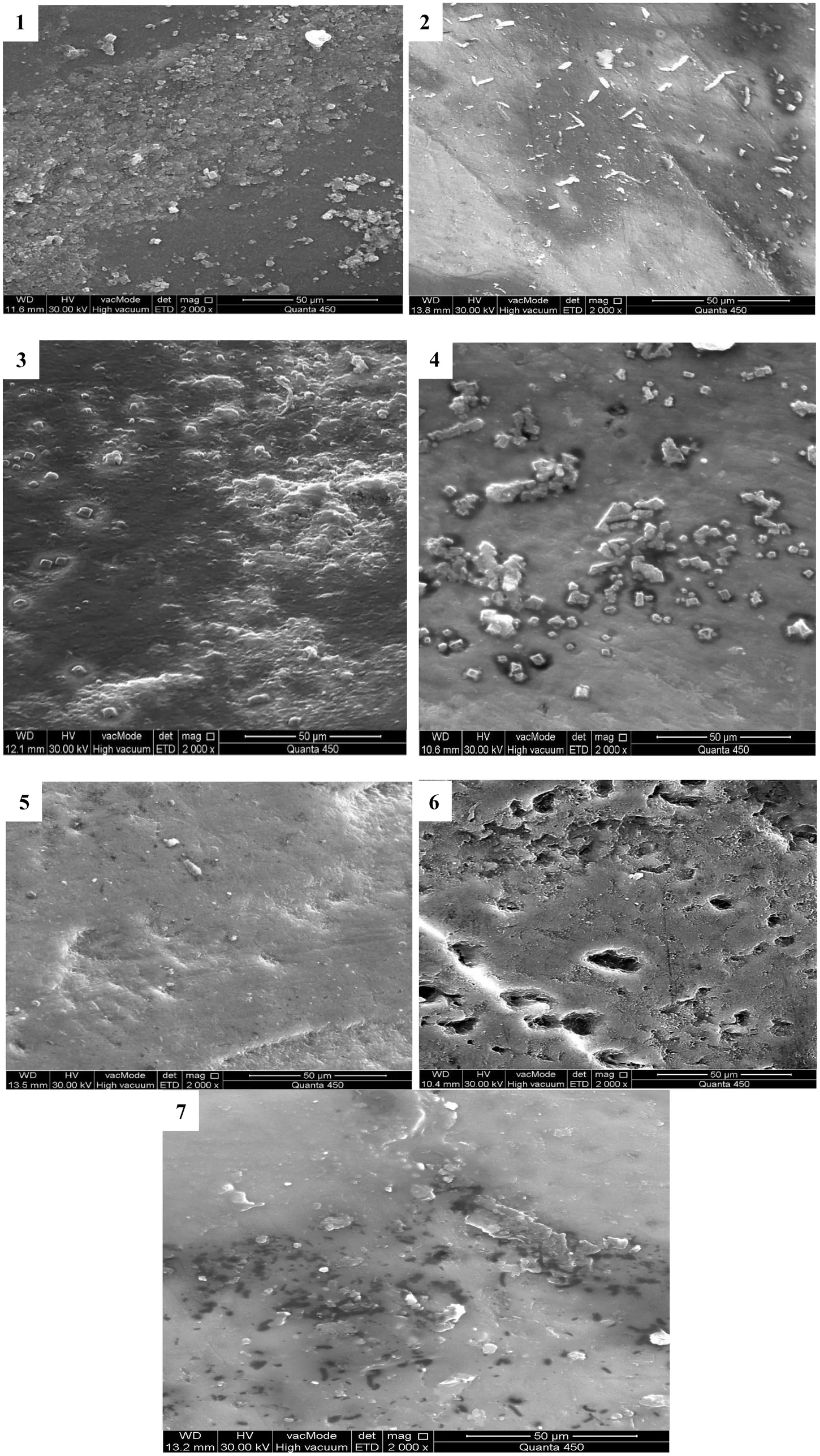
SEM image for enamel at 2,000× magnification. (1) enamel after application of FiB, (2) enamel after application of BoB, (3) enamel after application of BoT, and (4) enamel after application of TM indicated the accumulation of particles, well remineralized and smooth. (5) demineralized enamel (6) non-protected enamel, showing pores and component looses, and (7) sound enamel (untreated enamel).

SEM analysis at ×2,000 magnification revealed clear differences in enamel surface morphology among the study groups (Figure 7). The negative control group (demineralized enamel) presented severe surface degradation with pronounced irregularities and large deep pores, indicating significant mineral loss (Figure 7-5), but conversely, the enamel surfaces of the experimental groups (FiB, BoB, and BoT) were smoother with decreased porosity and clear mineral deposition. Crystals were observed in all treated groups, but with different morphologies: irregular crystal aggregates (FiB, Figure 7-1); larger cubic-like particles (BoB, Figure 7-2); and more uniform and well-organized crystalline structures (BoT, Figure 7-3), whereas the positive control group (Tooth Mousse) had a relatively smooth and homogeneous surface with minimal porosity, suggesting effective remineralization (Figure 7-4).

#### Atomic force microscopy (AFM)

**Figure 8:**
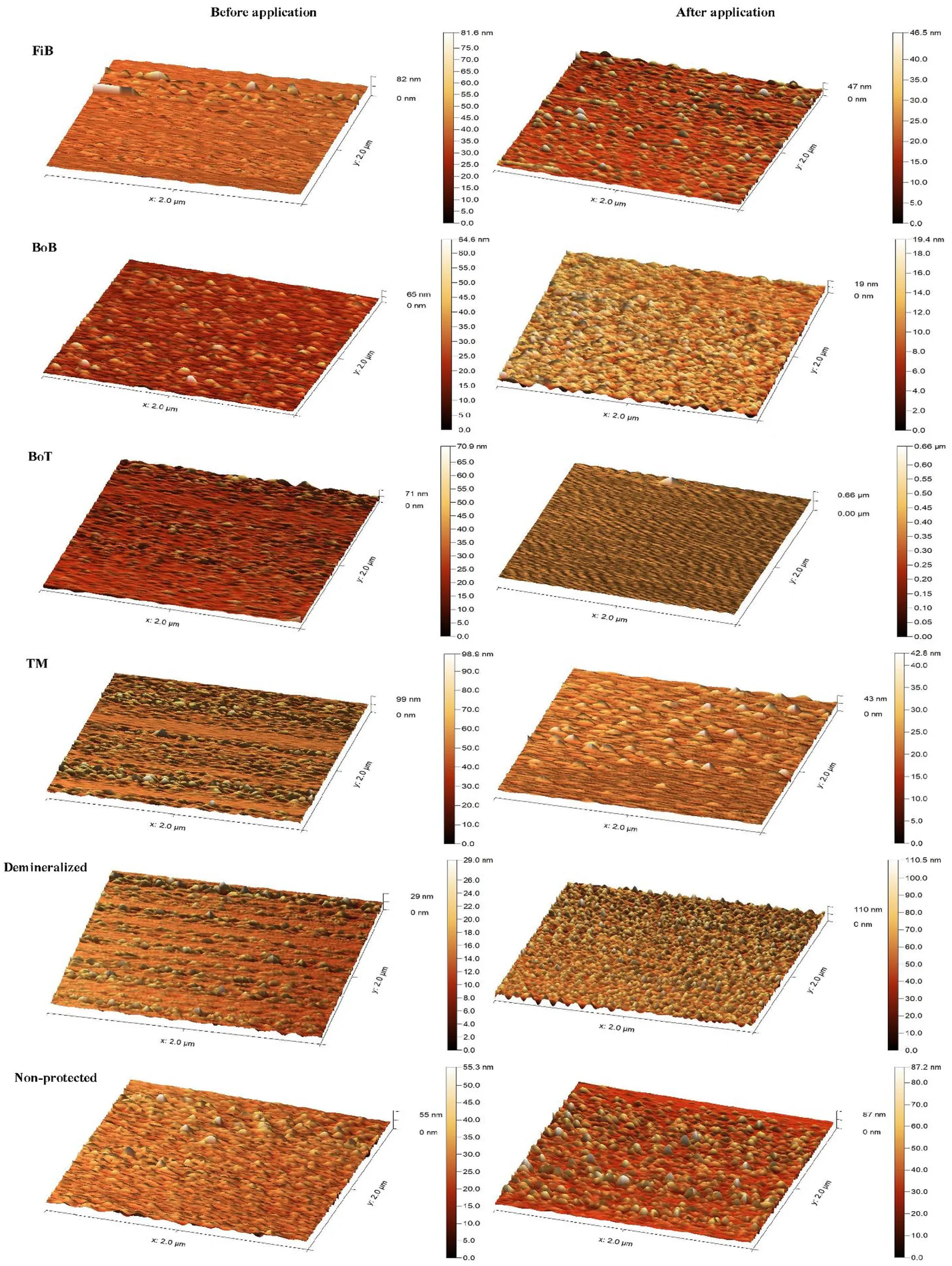
The three-dimensional AFM images (2 × 2 µm) of enamel surface roughness, obtained from five distinct areas, showed that the experimental materials had a favorable effect on enamel remineralization. Topographic AFM imaging revealed localized surface roughness before and after application of experimantal groups (FiB, BoB, and BoT) and positive control (TM) as well as two negative controls (Demineralization and non-protected enamels).

**Figure 9:**
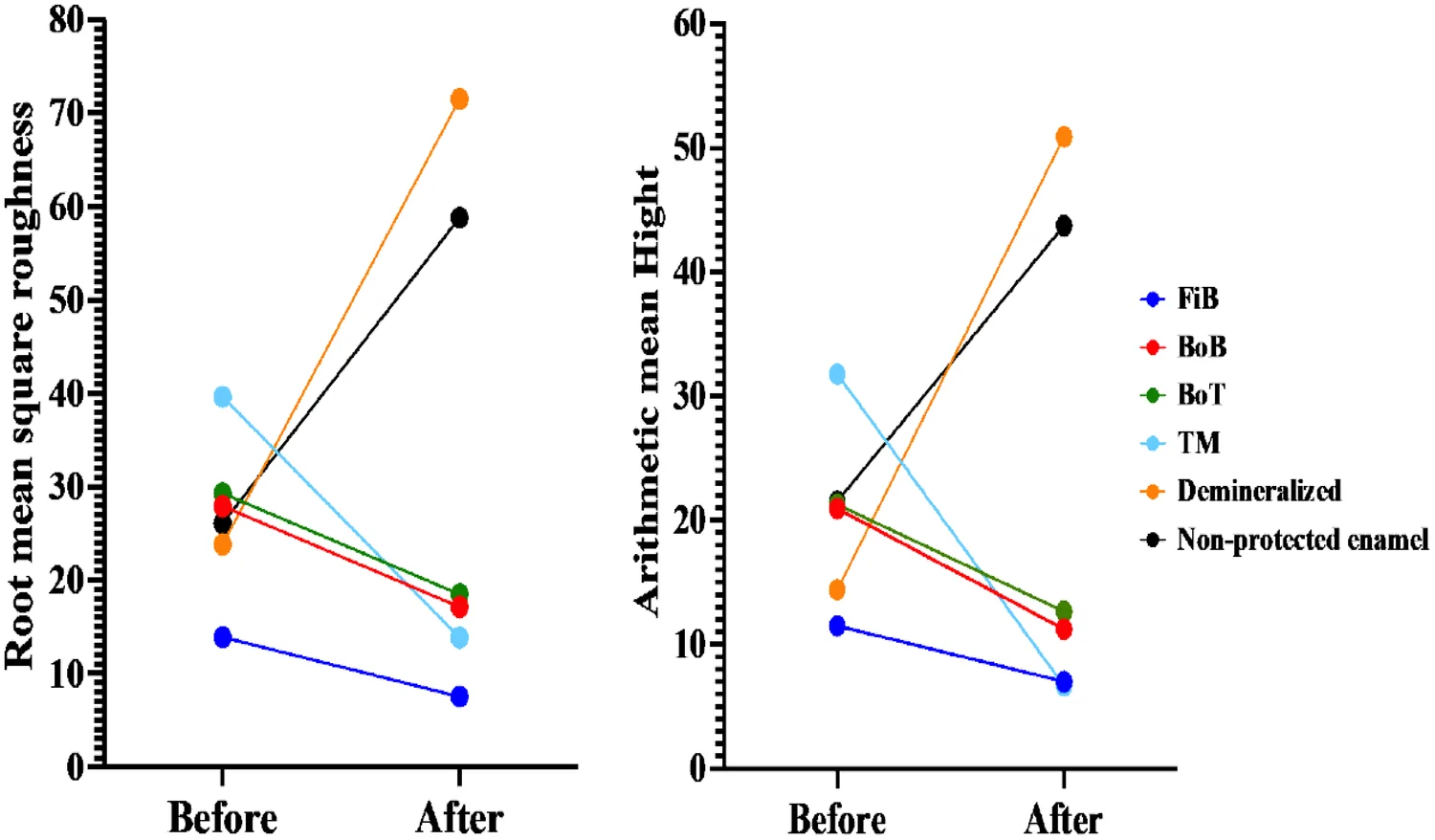
The roughness surface and arithmetic mean height for enamels before and after application of FiB, BoB, and BoT as experimental remineralization agents, tooth mousse paste (TM) as positive control, demineralized and non-protected enamel as negative control.

AFM analysis (Figure 8) demonstrated differences in enamel surface roughness before and after treatment across the study groups. In the FiB and BoB groups, surface topography appeared qualitatively smoother following treatment; however, insignificant differences were observed in root mean square roughness (Sq) or arithmetic mean height (Sa) (*p* > 0.05) (FiB; *p* = 0.19 and *p* = 0.18, and BoB; *p* = 0.11 and *p* = 0.35, respectively). In contrast, the BoT group showed a significant reduction in both Sq and Sa values (*p* = 0.03 and *p* = 0.04, respectively) after treatment (*p* < 0.05), indicating improved surface smoothness and homogeneity (Figure 9). The positive control (Tooth Mousse) exhibited a pronounced reduction in surface roughness, with significant decreases in both Sq and Sa (*p* < 0.05). For example, the mean Sq value decreased from 33.62 ± 12.3 nm to 10.2 ± 6.01 nm, accompanied by a reduction in peak-to-valley height across the scanned surface. Conversely, the non-protected enamel group demonstrated a statistical increase in surface roughness following demineralization (*p* < 0.05), with Sq values increasing from 23.83 ± 14.7 nm to 71.53 ± 54.2 nm. AFM topography revealed pronounced surface irregularities characterized by deeper valleys and sharper peaks (Figure 9).

## Discussion

The purpose of this study was to estimate the remineralizing potential of the three biomaterials (FiB, BoB and BoT) derived from natural waste, compared with a commercial agent (TM) using XRD, SEM and AFM, providing a comprehensive analysis of the structural, morphological and topographical changes in the treated enamel, and the complementary analytical methods used to support the reliability of the findings. Crystallite size is the key factor that can impact the solubility and ion release of calcium phosphate materials, and in the present study, BoT and BoB had smaller crystallite sizes (52.5 nm and 59.9 nm, respectively) than FiB (83.2 nm). Smaller crystallites are related to broader XRD peaks and higher FWHM values, which may indicate lower crystallinity and more surface defects, and such structural features increase the surface energy of the material and make it more reactive in an aqueous or acidic environment. Therefore, the materials with a smaller crystallite size and higher FWHM value are expected to dissolve more easily and release calcium and phosphate ions more rapidly, which is important for remineralisation (24).

The EDX measurements demonstrated marked differences in the mineral composition between the experimental groups, specifically, the FiB, BoB and BoT groups presented higher Ca concentrations than the negative control, indicating the active deposition of minerals on the enamel surface, and additionally, the P levels in these groups were considerably lower than those of the controls, resulting in an altered Ca/P ratio, which is a determining factor in the crystallographic stability of hydroxyapatite, the main mineral phase of the enamel (25, 26). SEM image analysis, are representative only of the visible two-dimensional surface pores in the analyzed micrographs, and should not be confused with the three-dimensional pore volume calculated by BET analysis, therefore, BET provides a more accurate quantitative description of the internal porous structure. In the present study, FiB and BoT showed a higher surface area (1-2 m^2^/g) compared to BoB (0.3 m^2^/g), which may allow more exposed surface sites for contact with the surrounding fluid, and this may facilitate the librate the calcium and phosphate ions from the surface of the material (24). So that the higher surface area of FiB and BoT may lead to a better remineralisation potential than BoB.

The observed changes in the mineral composition suggest that these biomaterials exert an active control over the remineralization process by promoting the selective incorporation of Ca, which might be due to the greater availability of calcium and the ion release kinetics of the calcined materials, however, these results did not differ from those of the TM or sound enamel, demonstrating similar efficacy in mineral restoration. Furthermore, chloride and oxygen content remained constant among the groups, which suggested that the treatments did not cause unwanted chemical changes but selectively altered the mineral matrix of the enamel.

The mesoporous structure of the samples, with a pore diameter in the range of 10–20 nm, suggests that the particles can be easily penetrated by the oral fluids, and therefore the calcium phosphate surface is more easily accessed, and as a result, creating a local environment favourable for mineral deposition. The SEM results can also support this interpretation because rough, irregular or aggregated particles can present more exposed surface sites for interaction with the surrounding medium, and additionally, the smaller particle sizes also result in an increased surface area-to-volume ratio. Therefore, taking into account the BET, SEM and particle size characteristics, the materials present a structure that may promote fluid access, ion transport and sustained calcium phosphate deposition.

SEM examination (2000x) allowed a detailed analysis of surface morphological changes, and enamel surfaces treated with FiB, BoB or BoT showed visible signs of remineralization, with precipitation of calcium phosphate crystals. The BoT group showed a significantly less porous surface texture, indicating more complete and efficient mineral infilling, which is in accordance with the observed higher crystallinity and structural homogeneity, and samples treated with BoB formed large and separate cubic crystals, which is indicative of an intermediate phase of remineralization, whereas FiB-treated surfaces showed scattered crystal formations, which is indicative of an early phase of mineral deposition (27). In contrast, the TM group showed a smooth and homogeneous surface with minimal porosity, which is indicative of an advanced state of remineralization, and the demineralized control samples showed larger, deeper pores, consistent with the substantial mineral loss from acid exposure (28). The findings of this study are in agreement with previously reported remineralization patterns using calcium phosphate-based systems, but there are limited studies on the surface roughness of enamel using such natural waste materials, and the application of different pastes resulted in protective and partially remineralized enamel (29, 30). AFM three-dimensional topographic imaging allowed for precise measurement of surface roughness changes, and statistical analysis of the Sq and Sa indicated that FiB and BoB treatments did not produce statistically significant alterations, although qualitative observation showed localized smoothing, whereas the BoT group showed statistically significant reductions in both Sq and Sa parameters, demonstrating a measurable improvement in surface homogeneity and smoothness. This implies that BoT has better physicochemical properties for effective surface remineralization, and the most pronounced effect was in the TM group, where statistically significant decreases in Sq and Sa were accompanied by a marked reduction in peak-to-valley height from 99 nm to 32 nm, indicating effective filling of surface defects and successful recovery of enamel topography (31). Untreated demineralized enamel showed a sharp increase in all roughness metrics, from a smooth baseline to a highly irregular morphology characterized by sharp peaks and deep valleys, thereby quantitatively confirming the erosive effect of acid exposure. The integrated results from XRD, SEM and AFM analyses collectively show that the biomaterials tested, especially BoT, promote enamel remineralization, as indicated by positive shifts in mineral composition, reduction in surface porosity and reduced topological roughness, although the commercial agent TM yielded the most advanced restorative outcomes, BoT presented itself as an effective natural alternative, with efficient mineral deposition and significant surface smoothing. These findings suggest that BoT is a strong candidate for sustainable and cost-effective remineralization methods, and the present results highlight the potential of biologically derived materials in developing novel strategies for enamel repair and caries prevention. According to our knowledge, there are few reports of the use of thermally calcined bovine bone and bovine teeth for human enamel remineralization, and on the other hand, there are some studies that reported that the isolated hydroxyapatite from fish bones in slurry or paste form enhances enamel mineralization and increases the enamel surface hardness (15, 32). The superior remineralisation potential of BoT could be attributed to its smaller crystallite size, mesoporous structure, relatively high surface area, lower thermal stability and calcium-deficient composition, which could lead to the faster release of calcium and phosphate ions, better interaction with demineralised dental tissues and more sustained mineral deposition, making BoT the most favorable candidate among the tested bio-waste materials for remineralisation applications. The results showed that the experimental materials listed can be effective for enamel remineralization, so the bovine bone and teeth for enamel remineralization and the calcined raw materials used for remineralization can be considered a strength of this study, which increases the scientific contribution and originality of the work. Several limitations of the study should be recognized, including that this study was performed under *in vitro* conditions, which do not reproduce all of the complex biological conditions of the oral environment, such as saliva composition, biofilm activity and dynamic pH changes, and the pH-cycling model, although standardized, cannot simulate long-term intraoral conditions, and in addition, this study focused mainly on physicochemical and surface analysis, but did not evaluate the mechanical properties, such as microhardness, wear resistance or bond strength, which are important for clinical performance. Moreover, the relatively wide and non-standardized particle sizes of the calcined materials could have influenced the remineralization, and the short experimental duration did not allow for the assessment of long-term stability and resistance to multiple acid challenges, and in addition, the absence of *in vivo* or clinical validation restricts the direct clinical application of these findings. The current study used broad particle sizes of calcined materials for enamel remineralization, and the production and application of nanoparticles, as well as the use of specific particle sizes, will be investigated in future studies, which should also consider *in vivo* conditions and long-term durability to further validate clinical applicability. This study's finding clearly showed that bioactive materials derived from natural waste sources, in particular BoT, were capable of effectively promoting enamel remineralization, and suggest that these materials represent a sustainable and promising alternative to conventional remineralization agents.

## Conclusion

The present study shows that thermally calcined biowaste materials (FiB, BoB and BoT) are able to promote enamel remineralization, as evidenced by the EDX, SEM and AFM combined analysis, and among the experimental groups, BoT resulted in the most marked increase in terms of mineral gain and surface smoothness, whereas FiB and BoB mostly reflected the initial stages of remineralization. As expected, the commercial agent Tooth Mousse showed the most pronounced reduction in surface roughness, and altogether, these results demonstrate that calcined biowaste can be converted into hydroxyapatite-like materials with a favorable Ca/P ratio that promote enamel mineral recovery. Moreover, these results point to the potential of natural, cost-effective and environmentally friendly materials as alternatives to conventional remineralizing agents, particularly for early stages of enamel demineralization management.

## Data Availability

The raw data supporting the conclusions of this article will be made available by the authors, without undue reservation.
